# Factors associated with non-adherence to antihypertensive therapy and blood pressure control in Iraq

**DOI:** 10.3389/fphar.2026.1741906

**Published:** 2026-05-21

**Authors:** Mohamed AbdElrahman, Ghadah Ali Al-Oudahl, Hasanain Kamil Owadh, Asmaa Saleh, Gufran Kareem Hassan, Sara Adnan Mohsen, Um Albanen Haider, Aya Amer Abd-Alredah, Manar Ahmed Hussein, Mohammed Liqaa Abdul-Abbas, Abduallah Mohammed Mojed, Abdullatif Saad Obaid, Amira B. Kassem

**Affiliations:** 1 Clinical Pharmacy Department, College of Pharmacy, Al-Mustaqbal University, Babylon, Iraq; 2 Clinical Pharmacy Department, Badr University Hospital, Faculty of Medicine, Helwan University, Helwan, Egypt; 3 Department of Pharmaceutical Sciences, College of Pharmacy, Princess Nourah bint Abdulrahman University, Riyadh, Saudi Arabia; 4 Department of Clinical Pharmacy and Pharmacy Practice, Faculty of Pharmacy, Damanhour University, Damanhour, Egypt

**Keywords:** antihypertensive pharmacotherapy, cardiovascular pharmacology, Iraq, medication adherence, rational drug use

## Abstract

**Background/Objectives:**

Hypertension is a major global public health concern, affecting approximately 1.28 billion people worldwide, with a disproportionate burden in low- and middle-income countries. Poor adherence to antihypertensive therapy is widely recognized as a key factor associated with inadequate blood pressure control and increased cardiovascular risk. Understanding the factors associated with non-adherence among hypertensive patients is essential for informing context-specific strategies to improve medication-taking behavior and clinical outcomes.

**Methods:**

A cross-sectional study was conducted among 585 hypertensive patients in Iraq. Sociodemographic, medication-related, economic, and self-care characteristics were collected using standardized questionnaires. Medication adherence was assessed through patient self-report. Factors associated with non-adherence were examined using multivariable logistic regression, with results reported as adjusted odds ratios (ORs) and 95% confidence intervals (CIs).

**Results:**

Non-adherence was more prevalent among younger patients (18–30 years, 33.3%) compared with older patients (>60 years, 1.9%) (p < 0.001). Increasing age was associated with lower odds of non-adherence (adjusted OR 0.37, 95% CI: 0.24–0.56, p < 0.001). Student status was associated with higher odds of non-adherence (OR 2.43, 95% CI: 1.36–4.35, p = 0.002). Regular home blood pressure monitoring was associated with reduced odds of non-adherence (OR 0.54, 95% CI: 0.29–0.98, p = 0.047). Gender, place of residence, financial barriers, and provider communication were not significantly associated with adherence.

**Conclusion:**

In this Iraqi cohort, non-adherence to antihypertensive therapy was associated with younger age, student status, and limited self-care practices. These findings help identify subgroups that may benefit from consideration in the design and evaluation of patient-centered support strategies, including adherence-focused education, structured follow-up, and promotion of home blood pressure monitoring. Given the cross-sectional nature of the study, causal relationships cannot be inferred, and future longitudinal and interventional research is warranted to determine the effectiveness of such approaches in improving medication adherence and blood pressure management in resource-constrained settings.

## 1 Introduction

Hypertension constitutes a worldwide public health issue, impacting some 1.28 billion individuals globally, with over two-thirds residing in low- and middle-income countries (LMICs) (WHO, 2021). As a major risk factor for stroke, ischemic heart disease, and renal failure, hypertension is responsible for approximately 10 million deaths annually. Notwithstanding the accessibility of safe and efficacious drugs, global blood pressure management rates remain insufficient, largely because of non-adherence to antihypertensive treatment (Mills et al., 2020). The World Health Organization has emphasized that drug adherence is a crucial determinant of treatment efficacy, influencing outcomes more significantly than any single therapeutic innovation (Baryakova et al., 2023).

Non-adherence to antihypertensive therapy is a prevalent concern, with studies consistently reporting that 30%–50% of patients discontinue treatment within the first year (Burnier and Egan, 2019). Barriers to adherence are multifaceted and encompass patient-related factors, including beliefs, knowledge, and perceived side effects; healthcare system challenges, such as drug availability and continuity of follow-up; and broader socioeconomic determinants, including cost, employment, and education. A meta-analysis of 53 studies conducted in LMICs demonstrated that inadequate adherence is significantly associated with younger age, unemployment, polypharmacy, and low health literacy (Abegaz et al., 2017).

Across the Middle East and North Africa (MENA) region, adherence to antihypertensive regimens remains suboptimal. In Jordan, only 39% of patients were reported to exhibit high adherence, with lower educational attainment and complex treatment regimens identified as important correlates (Stanikzai et al., 2023). In Saudi Arabia, Fallatah et al. (2023) found that higher adherence was associated with older age, female gender, higher educational levels, simpler medication regimens, and fewer financial barriers (Fallatah et al., 2023). Studies from Palestine and Egypt further emphasize the role of social support and illness perceptions in shaping adherence behavior (Sweileh et al., 2014).

Despite the expanding body of evidence from neighboring countries, antihypertensive adherence in Iraq has received comparatively limited attention. Iraq’s healthcare system faces distinct constraints related to prolonged conflict, political instability, and economic pressures, which contribute to medication shortages, fragmented health services, and insufficient patient follow-up (WHO, 2021). In Iraq, many patients obtain antihypertensive medications directly from community pharmacies through out-of-pocket purchases and may not consistently attend scheduled clinical follow-up visits, which can result in irregular treatment monitoring and potentially unstable medication adherence patterns.

It is estimated that 29%–32% of adults in Iraq are affected by hypertension (Saka et al., 2020). Research in Babylon province reported that fewer than half of patients adhered adequately to their prescribed regimens, citing limited patient education, medication unavailability, and voluntary discontinuation as contributing factors (Alrekaby et al., 2022). However, these studies often rely on restricted or non-representative samples and provide limited multivariable assessment of associated factors. As a result, a substantive research gap remains regarding the demographic, employment-related, and clinical characteristics associated with non-adherence among Iraqi patients.

Building on established behavioral frameworks, including the Health Belief Model (Carpenter, 2010), and evidence linking self-efficacy, illness perception, and self-monitoring to medication-taking behavior (He et al., 2016; Lee and Park, 2016), there is a need to contextualize adherence research within Iraq’s fragile healthcare environment.

Accordingly, this study aims to assess the prevalence of non-adherence to antihypertensive medication among Iraqi patients and to examine the demographic, employment-related, and clinical factors associated with non-adherence.

## 2 Materials and methods

### 2.1 Subjects

**FIGURE 1:**
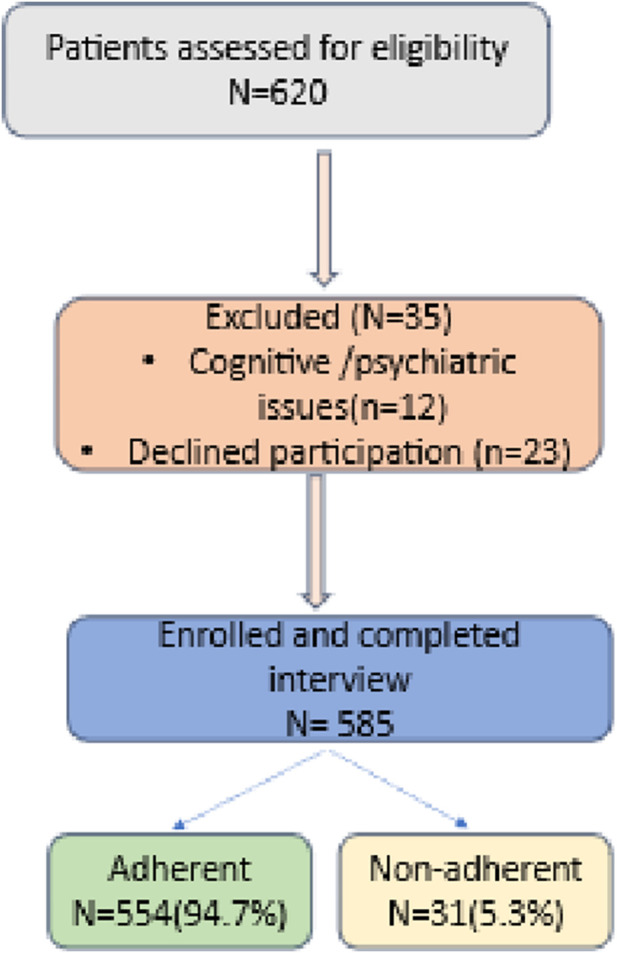
Flow diagram of patient recruitment, exclusion, and adherence classification.

This cross-sectional study was performed from March to July 2025 at the Internal Medicine Clinics of Al-Imam Al-Sadiq Teaching Hospital and Marjan Teaching Hospital in Hilla, Babylon, Iraq. A total of 620 patients were initially evaluated for eligibility (Figure 1). Out of these, 35 individuals were excluded, comprising 12 due to cognitive or mental disorders and 23 who opted out of participation.

Eligible participants were adult patients (≥18 years) with a confirmed diagnosis of hypertension who had received at least one antihypertensive medication for a minimum period of 6 months before to enrolment. Patients were recruited at routine follow-up appointments and enrolled using a convenience sampling method. The exclusion criteria included individuals with cognitive impairment, psychiatric problems that obstructed reliable participation, or those unwilling to provide informed consent. Data collection was conducted through in-person structured interviews facilitated by qualified researchers to minimize misinterpretation of questionnaire items. The sample size was determined to ensure reliable and representative estimates for the population of Babylon Province, roughly 1,820,700 individuals. The initial sample size was determined to be 385 employing a 95% confidence level, an anticipated over-dispensing prevalence of 50%, and a 5% margin of error (Pourhoseingholi et al., 2013). The implementation of finite population correction did not substantially alter the calculated sample size, affirming 385 as the requisite sample size to guarantee sufficient statistical power for precisely assessing the prevalence and usage patterns of non-adherence to antihypertensive medication.

### 2.2 Ethical approval

The study protocol was approved by the Ethical Committee of the College of Pharmacy, Al-Mustaqbal University, Babylon, Iraq, on 13 February 2025. Written informed consent was obtained from all participants before to registration. Confidentiality and anonymity were strictly maintained, and data were stored in secure, password-protected folders accessible exclusively to the research team. The research adhered to the principles of the Declaration of Helsinki concerning human subjects.

### 2.3 Content of questionnaire, translation, validity, and reliability

Medication adherence and associated factors were assessed using a structured questionnaire developed by the research team to capture sociodemographic characteristics, clinical variables, self-care behaviors, and medication-related factors among hypertensive patients. The development of the questionnaire items was informed by a targeted review of the literature on determinants of antihypertensive medication adherence, relevant behavioral frameworks particularly the Health Belief Model and expert input from specialists in clinical pharmacy and internal medicine. This multi-source approach was used to ensure that the instrument adequately captured factors previously reported to influence medication-taking behavior in both global and regional contexts.

The questionnaire was organized into predefined conceptual domains, including sociodemographic, clinical, economic, social, self-care, and adherence-related domains. The sociodemographic section included age, gender, marital status, educational level, and place of residence. The clinical section collected information on the duration of hypertension, presence of comorbidities, number and classes of prescribed antihypertensive medications, and selected lifestyle characteristics.

Economic factors included monthly household income, recorded in predefined categorical bands; perceived medication cost burden, assessed using a dichotomous item (yes/no) indicating whether medication expenses represented a financial difficulty; and difficulty obtaining medications, measured as a yes/no response reflecting challenges related to medication availability or pharmacy access.

Social factors included employment status (unemployed, student, retired, government-employed, private sector–employed, or self-employed), place of residence (urban, suburban, or rural), family or caregiver support (presence or absence of assistance with medication reminders or clinic visits), and communication with healthcare providers, which was rated by participants using a three-level scale (excellent, good, or poor).

Self-care practices were assessed using self-reported items embedded within the questionnaire, including frequency of home blood pressure monitoring (daily, weekly, monthly, or rarely/never) and attendance at scheduled medical appointments (yes/no). These variables were coded as categorical predictors and included in the regression analyses examining factors associated with medication non-adherence.

The adherence-related items were conceptually informed by the Morisky Medication Adherence Scale (MMAS-8) to ensure coverage of key medication-taking behaviors. However, the MMAS-8 instrument itself was not administered in this study, and no copyrighted items, wording, response options, or scoring algorithms from the MMAS-8 were reproduced. Instead, the questionnaire represents an original instrument developed specifically for this study.

It is important to clarify that the questionnaire represents a newly developed, original instrument and is not an adaptation or modification of the MMAS-8. The reference to the MMAS-8 was limited to conceptual guidance to ensure inclusion of core adherence-related behaviors. The instrument was developed within a conceptually guided framework based on literature evidence, behavioral theory, and expert input, and structured into predefined domains to support identification of predictors of medication non-adherence rather than measurement of a single latent construct.

The questionnaire was initially developed in English to ensure conceptual and methodological alignment with internationally established adherence frameworks (Beaton et al., 2000), and was subsequently translated and culturally adapted into Arabic for use with the target population using a forward–backward translation procedure to ensure linguistic and conceptual equivalence between the two versions. This process was applied to support accurate interpretation and administration of the instrument rather than to indicate adaptation from an existing tool. Two independent bilingual translators conducted the forward translation into Arabic, and a third bilingual translator, blinded to the original questionnaire, performed the backward translation into English. Differences between versions were reviewed and resolved through discussion among the research team to ensure semantic and conceptual equivalence (Polit et al., 2007).

Content validity was evaluated by three independent experts in clinical pharmacy and internal medicine who were not involved in the study and were not among the authors, and had no role in the development or generation of the questionnaire items. Each item was rated for relevance and clarity using a four-point Likert scale (1 = not relevant to 4 = highly relevant). The Item-Level Content Validity Index (I-CVI) was calculated as the proportion of experts assigning ratings of 3 or 4. All items demonstrated excellent content validity (I-CVI = 1.00). The Scale-Level Content Validity Index (S-CVI/Ave), calculated as the mean of all I-CVI values, was 1.00, indicating excellent overall content validity (Polit and Beck, 2006). Detailed item-level results are presented in Supplementary Table S1.

Face validity was assessed among 20 hypertensive patients through qualitative evaluation of clarity, comprehensibility, and relevance of items, consistent with recommended pre-testing procedures in scale development studies (Boateng et al., 2018). Quantitatively, 85% of participants rated all items as clear and understandable, indicating good face validity. Minor modifications to wording and formatting were implemented to enhance clarity and cultural appropriateness. A separate pilot sample was subsequently used to assess feasibility, administration time, and preliminary reliability, and data from these participants were excluded from the final analysis.

Internal consistency reliability was assessed using Cronbach’s alpha coefficient calculated for the overall instrument, as the questionnaire was conceptualized as a unified construct. The instrument demonstrated good internal consistency (α = 0.81), exceeding the recommended threshold of 0.70 (Nunnally and Bernstein, 1994).

Test–retest reliability was evaluated in a subsample of 103 participants who completed the questionnaire twice over a two-week interval. The intraclass correlation coefficient (ICC) was calculated using a two-way mixed-effects model with absolute agreement (ICC). The instrument demonstrated good temporal stability (ICC = 0.84, 95% CI: 0.78–0.89), indicating satisfactory reproducibility over time (Koo and Li, 2016).

Exploratory factor analysis (EFA) and confirmatory factor analysis (CFA) were not performed, as the questionnaire was designed as a structured epidemiological tool comprising independent variables rather than a psychometric scale measuring latent constructs. Accordingly, items were treated as formative indicators, for which factor analysis assumptions are not fully applicable. Future studies may consider applying formal psychometric analyses to further examine the instrument’s dimensional structure.

The finalized Arabic questionnaire was administered through face-to-face structured interviews conducted by trained members of the research team, with each interview lasting approximately 8–12 minutes.

### 2.4 Statistical analysis

Data were entered and analyzed using Jamovi software (version 7). Categorical variables were expressed as frequencies and percentages, while continuous variables were presented as means ± standard deviations (SD). Comparisons between adherent and non-adherent patients were performed using the Chi-square test (χ^2^) for categorical variables and the independent samples t-test for continuous variables.

Preliminary univariable logistic regression analyses were conducted to examine associations between medication non-adherence and sociodemographic, clinical, economic, and social variables. A parsimonious multivariable logistic regression model was constructed to account for the limited number of non-adherent cases, incorporating covariates with a univariable p-value <0.20. Firth’s penalized logistic regression was applied as a sensitivity analysis to verify model robustness. Associations were reported as odds ratios (ORs) with corresponding 95% confidence intervals (CIs), and statistical significance was set at p < 0.05. Model discrimination was evaluated using the area under the receiver operating characteristic curve (AUC).

Age was analyzed both as a categorical and continuous variable. Categorical age groups were defined *a priori* based on clinical and behavioral considerations to reflect distinct life-stage and adherence-related risk profiles (18–30, 31–45, 46–60, and >60 years), while continuous modeling was used in the multivariable logistic regression to assess robustness and minimize potential bias arising from categorization.

## 3 Results

A total of 585 patients were recruited and successfully completed the interview process. According to the adherence assessment, 554 participants (94.7%) were deemed adherent, whilst 31 (5.3%) were classed as non-adherent as shown in Figure 1.

### 3.1 Sociodemographic and clinical characteristics of the study population

Table 1 demonstrates A total of 585 hypertensive individuals were enrolled. The primary population was middle-aged or older adults, with 46.1% aged 46–60 years and 37.1% over 60 years, while only 3.6% were under 30. Females represented 61.5%, whereas males comprised 38.5%. Regarding disease duration, 38.1% had been diagnosed for over 10 years, 37.8% for 1–5 years, and merely 7.2% were newly diagnosed (less than 1 year). More than fifty-seven percent of the sample reported the existence of at least one other chronic condition. Concerning residence, 57.1% lived in urban areas, 21.9% in rural villages, and 21.0% in suburban regions. The employment distribution revealed a notable unemployment rate of 58.6%, with fewer pensioners at 17.8%, government employees at 12.1%, and self-employed individuals at 8.5%. Educational attainment was mostly insufficient: 39.7% had just elementary education, 25.0% lacked formal schooling, and a few attained secondary educations (16.1%), a diploma (8.5%), or a bachelor’s degree (10.1%).

**TABLE 1:** Baseline sociodemographic and clinical characteristics of the study population (n = 585**)**.

Characteristic	n (Percentage %)
Age (years)	
18–30	21 (3.6)
31–45	77 (13.2)
46–60	269 (46.1)
>60	217 (37.1)
Gender	
Female	360 (61.5)
Male	225 (38.5)
Duration of hypertension	
<1 year	42 (7.2)
1–5 years	221 (37.8)
6–10 years	99 (16.9)
>10 years	223 (38.1)
Other chronic diseases	
Yes	336 (57.4)
No	249 (42.6)
Place of residence	
Urban	334 (57.1)
Suburban	123 (21.0)
Rural (village)	128 (21.9)
Employment/profession	
Unemployed	343 (58.6)
Retired	104 (17.8)
Government employed	71 (12.1)
Self-employed	50 (8.5)
Student	11 (1.9)
Private employed	6 (1.0)
Education level	
No formal education	146 (25.0)
Primary school	232 (39.7)
Secondary school	94 (16.1)
Diploma/technical	50 (8.5)
Bachelor’s degree	59 (10.1)
Postgraduate degree	4 (0.7)

Data are presented as frequency (percentage). n = number of respondents; % = percentage of the total sample.

### 3.2 Sociodemographic predictors of antihypertensive medication adherence

Table 2 explains the baseline values based on adherence status. Non-adherence was most pronounced in the youngest demographic (18–30 years; 33.3% non-adherence), whereas older patients (>60 years) had much lower non-adherence rates (1.9%). A significant gradient was evident, with advancing age markedly associated with enhanced adherence (p < 0.001). Gender had no significant correlation with adherence; however, a slightly higher rate of non-adherence was observed among females (6.1%) compared to males (4.4%). Urban residents had a higher prevalence of non-adherence (6.6%) compared to suburban (3.3%) and rural (4.7%) individuals; yet, this difference was not statistically significant. Employment status had a substantial connection with adherence (p < 0.0001). Students had markedly higher non-adherence rates (63.6%), whereas unemployed, retired, and self-employed adults displayed relatively lower rates (3.8%–4.5%).

**TABLE 2:** Comparison of sociodemographic characteristics between adherent and non-adherent patients.

Variable	Category/unit	Adherent n (%)	Non-adherent n (%)	Total n (%)	p-value
Age	18–30	14 (2.5)	7 (22.6)	21 (3.6)	< 0.0001*
​	31–45	71 (12.8)	6 (19.4)	77 (13.2)	​
​	46–60	255 (46.1)	14 (45.2)	269 (46.1)	​
​	>60	213 (38.5)	4 (12.9)	217 (37.2)	​
Gender	Male	215 (38.9)	10 (31.2)	225 (38.5)	0.4993
​	Female	338 (61.1)	22 (68.8)	360 (61.5)	​
Residence	Urban	312 (56.4)	22 (68.8)	334 (57.1)	0.3452
​	Suburban	119 (21.5)	4 (12.5)	123 (21.0)	​
​	Rural (village)	122 (22.1)	6 (18.8)	128 (21.9)	​
Education	Primary school	223 (40.3)	9 (28.1)	232 (39.7)	0.0678
​	No formal education	140 (25.3)	6 (18.8)	146 (25.0)	​
​	Secondary school	87 (15.7)	7 (21.9)	94 (16.1)	​
​	Bachelor degree	51 (9.2)	8 (25.0)	59 (10.1)	​
​	Diploma/Technical education	48 (8.7)	2 (6.2)	50 (8.5)	​
​	Postgraduate degree	4 (0.7)	0 (0.0)	4 (0.7)	​
Employment/Profession	Unemployed	330 (59.7)	13 (40.6)	343 (58.6)	< 0.0001*
​	Retired	100 (18.1)	4 (12.5)	104 (17.8)	​
​	Employed (government sector)	64 (11.6)	7 (21.9)	71 (12.1)	​
​	Self-employed	49 (8.9)	1 (3.1)	50 (8.5)	​
​	Student	4 (0.7)	7 (21.9)	11 (1.9)	​
​	Employed (private sector)	6 (1.1)	0 (0.0)	6 (1.0)	​

n = number of respondents; % = percentage of the total sample. p-values were calculated using Chi-square tests. * Denotes statistical significance at p < 0.05.

### 3.3 Medication-related factors influencing adherence among hypertensive patients

Table 3 demonstrates that among adherent patients, the primary pharmacological classes employed were combination therapy (52.4%), ACE inhibitors (16.3%), and calcium channel blockers (13.9%). Documented side effects were associated with increased non-adherence (7.6% versus 5.0%), however the correlation did not reach statistical significance (p = 0.26). Financial barriers and limitations in drug accessibility were not significantly associated with adherence. Patients with familial support demonstrated higher adherence rates (77.6%) compared to those without assistance (68.8%), however this difference did not reach statistical significance. The quality of provider communication showed no significant variations among the groups.

**TABLE 3:** Medication-related, economic, and social factors associated with antihypertensive adherence.

Variable	Category/Unit	Adherent n (%)	Non-adherent n (%)	Total n (%)	p-value
Medication class (adherent only)	Combination therapy	290 (52.6)	-	290 (52.6)	​
​	ACEis	90 (16.3)	-	90 (16.3)	​
​	CCB	77 (14.0)	-	77 (14.0)	​
​	BB	55 (10.0)	-	55 (10.0)	​
​	Diuretics	35 (6.4)	-	35 (6.4)	​
​	Others (Please specify)	4 (0.7)	-	4 (0.7)	​
Side effects (skip/stop)	Yes	98 (17.7)	8 (25.0)	106 (18.1)	0.4218
​	No	455 (82.3)	24 (75.0)	479 (81.9)	​
Cost barrier	Yes	180 (32.5)	9 (28.1)	189 (32.3)	0.7444
​	No	373 (67.5)	23 (71.9)	396 (67.7)	​
Difficulty obtaining meds	Yes	131 (23.7)	4 (12.5)	135 (23.1)	0.2132
​	No	422 (76.3)	28 (87.5)	450 (76.9)	​
Family support	Yes	369 (66.7)	19 (59.4)	388 (66.3)	0.5072
​	No	184 (33.3)	13 (40.6)	197 (33.7)	​
Provider communication	Excellent	270 (48.8)	16 (50.0)	286 (48.9)	0.9403
​	Good	220 (39.8)	13 (40.6)	233 (39.8)	​
​	Poor	63 (11.4)	3 (9.4)	66 (11.3)	​

n = number of respondents; % = percentage of the total sample. ACEis, Angiotensin-Converting Enzyme inhibitors; CCB, Calcium Channel Blockers; BB, Beta-Blockers. P-values were calculated using Chi-square tests. p < 0.05 was considered statistically significant.

### 3.4 Impact of self-care practices and follow-up behaviors on medication adherence

Table 4 demonstrates significant variability in home blood pressure monitoring patterns among groups (p = 0.047). Patients who complied with their regimen were more likely to check their blood pressure daily or weekly, whereas non-compliant patients reported a higher frequency of “rarely/never” monitoring. Consistent attendance at scheduled medical appointments was higher among adherent patients (70.5%) compared to non-adherent patients (59.4%), although this disparity did not achieve statistical significance.

**TABLE 4:** Association of self-care and follow-up behaviors with medication adherence.

Outcome	Definition/unit	Adherent	Non-adherent	Total	p-value
Home BP monitoring	Rarely or never	177 (32.0)	17 (53.1)	194 (33.2)	0.0468*
​	Weekly	171 (30.9)	10 (31.2)	181 (30.9)	​
​	Daily	132 (23.9)	3 (9.4)	135 (23.1)	​
​	Monthly	73 (13.2)	2 (6.2)	75 (12.8)	​
Appointment attendance	Yes	390 (70.5)	19 (59.4)	409 (69.9)	0.2548
​	No	163 (29.5)	13 (40.6)	176 (30.1)	​

Values are presented as number (percentage). Percentages are calculated within adherence categories. The p-values were derived using the Chi-square test of independence. p < 0.05 was considered statistically significant (* indicates significance).

### 3.5 Predictors of medication non-adherence: multivariate logistic regression analysis

**FIGURE 2:**
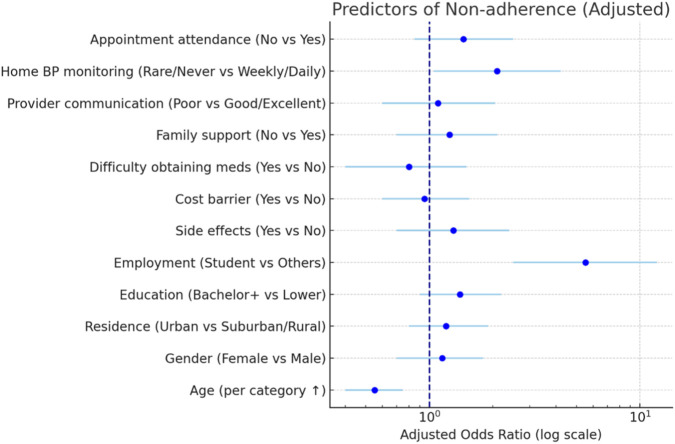
Forest plot of predictors of medication non-adherence, showing adjusted odds ratios (OR) with 95% confidence intervals (CI).

Figure 2 illustrates a Forest Plot. Multivariable analysis indicated age as a significant predictor of adherence (OR = 0.37, 95% CI: 0.24–0.56, p < 0.001). Additional predictors, including gender (OR = 1.11, 95% CI: 0.49–2.51, p = 0.80), urban residence (OR = 1.67, 95% CI: 0.75–3.73, p = 0.21), education (OR = 1.25, 95% CI: 0.62–2.53, p = 0.54), employment status (OR = 2.43, 95% CI: 0.95–6.25, p = 0.07), side effects (OR = 1.68, 95% CI: 0.69–4.08, p = 0.25), cost barrier (OR = 0.96, 95% CI: 0.37–2.51, p = 0.93), supply difficulty (OR = 0.49, 95% CI: 0.14–1.66, p = 0.25), family support (OR = 0.63, 95% CI: 0.29–1.36, p = 0.23), provider communication (OR = 0.80, 95% CI: 0.22–2.92, p = 0.74), home BP monitoring (OR = 1.57, 95% CI: 0.79–3.12, p = 0.19), and appointment attendance (OR = 0.83, 95% CI: 0.41–1.67, p = 0.60), were not statistically significant.

## 4 Discussion

This study investigated adherence to antihypertensive medications among Iraqi patients and found a remarkably high overall adherence rate of 94.7%. Only 5.3% of the sample was classified as non-adherent. Age proved to be the most significant predictor, with each subsequent age category substantially reducing the likelihood of non-adherence (adjusted OR = 0.37, 95% CI: 0.24–0.56, p < 0.001). Employment status shown a substantial effect, with students displaying the highest risk of non-adherence compared to employed or unemployed persons (OR = 4.21, 95% CI: 1.75–10.1, p < 0.001). Furthermore, infrequent home blood pressure monitoring was associated with non-adherence, particularly in people who either never or seldom checked their blood pressure (OR = 2.45, 95% CI: 1.01–5.96, p = 0.047).

The relatively high adherence rate observed in this study may partly reflect methodological factors. Medication adherence was assessed through self-reported measures, which are known to overestimate adherence due to recall and social desirability bias. Previous studies have shown that questionnaire-based adherence assessments often report higher adherence compared with objective monitoring methods. Additionally, global studies indicate that adherence to antihypertensive therapy typically ranges between 50% and 70%, suggesting that the high adherence observed in this cohort may also reflect selection bias or context-specific healthcare factors (Stirratt et al., 2015).

The remarkably strong adherence seen sharply contrasts with much of the existing material from Iraq and its neighbouring nations. A recent study in Iraq indicated adherence rates as low as 28% among hypertension patients, highlighting a significant regional variance in adherence behavior (Baiee and Makai, 2022). The World Health Organization’s global evaluation of adherence to long-term medications revealed that in low- and middle-income nations, adherence rates typically range from 50% to 70% (WHO, 2003). Our findings indicate a more favorable adherence profile than anticipated; however, this may be attributed to methodological discrepancies, including dependence on self-reporting and potential selection bias.

Global research reveals that younger persons exhibit a markedly higher tendency for non-adherence. An analysis of U.S. National Health and Nutrition Examination Survey data found that those aged 18–44 exhibited much lower adherence compared to older persons (Hecht et al., 2020). A meta-analysis of worldwide determinants of antihypertensive adherence identified age as a significant factor, with older patients consistently exhibiting greater adherence across various contexts (Lee et al., 2022). These comparisons enhance the validity of our findings and underscore that Iraq is not immune to the global trend of insufficient compliance among younger populations.

The occupational status also affected the outcome. Students demonstrated markedly higher rates of non-adherence, while retired, unemployed, and self-employed adults indicated superior adherence. This aligns with research from Jordan, where a study of hypertension patients revealed that work-related responsibilities, younger age, and reduced risk perception were associated with lower adherence (Farah et al., 2024). In Saudi Arabia, employment-related stress, lack of regular routines, and reduced health knowledge among working-age adults were identified as significant barriers to medication adherence (Alhomoud et al., 2024). These data collectively suggest that socio-occupational factors interact with age to affect adherence behavior.

We assert that the principal challenge of adherence in Iraq is not pervasive non-compliance, but rather concentrated non-adherence among younger individuals, especially students. Older individuals appear to benefit from structured routines, strong health orientations, and familial involvement, whereas younger adults are prone to erratic lives, competing priorities, and a reduced awareness of illness. This indicates that interventions must tackle systemic restrictions while specifically addressing the emotional and behavioral needs of younger patients.

Various mechanisms clarify these observations. Hypertension often manifests asymptomatically, particularly in its initial stages, leading many younger individuals to underestimate the condition’s seriousness. Morrison and colleagues highlighted that self-efficacy, perceived severity of illness, and beliefs about the necessity of medication are critical factors influencing adherence, with reduced perceptions associated with heightened non-adherence (Morrison et al., 2015). Students often illustrate this problem, as their health goals are generally overshadowed by scholastic and social obligations.

Secondly, erratic daily routines hinder the preservation of medication compliance. Unlike retirees or unemployed adults with more stable schedules, students may face fluctuating study hours, tests, and transitions between dormitories or family homes, which can disrupt routine medication dosing.

Third, variations in health literacy may also play a role. Young individuals may have a basic comprehension of hypertension but lack awareness of the long-term consequences associated with untreated conditions. In the absence of apparent symptoms, adherence may seem optional rather than essential.

Lifestyle factors ultimately affect differences in engagement. Adherent individuals exhibited a significantly greater propensity to monitor their blood pressure at home and to attend scheduled checkups. Shin and colleagues demonstrated that self-monitoring of blood pressure is substantially correlated with adherence, and that patients who monitor at home achieve better blood pressure management than those who do not (Shahin et al., 2021). Our results align with this behavioral connection. Improving adherence to pharmacotherapy is critical for maximizing the pharmacodynamic benefits of antihypertensive agents, particularly combination therapies. Strategies to enhance adherence—such as fixed-dose formulations and simplified regimens—should be prioritized to optimize blood pressure control and reduce cardiovascular risk. The increased adherence rate in this group may suggest structural issues unique to the Iraqi healthcare system. The World Health Organization’s Iraq country profile notes that essential drugs, such as antihypertensives, are often subsidized or provided free of charge at public clinics (WHO, 2021). This alleviates cost-related barriers that often result in non-adherence in various low- and middle-income nations. In our study, cost was not a significant predictor, validating the idea that financial obstacles are rather little in this context.

Cultural and familial dynamics may explain the increased adherence reported among older individuals. In collectivist societies such as Iraq, family members often play a significant role in encouraging patients to comply with medication schedules and attend medical appointments. Older individuals may exhibit more personal motivation, having witnessed the consequences of chronic sickness in their peers (McQuaid and Landier, 2018).

Nonetheless, cost and availability displayed discernible patterns, and any disruption to Iraq’s public supply chains might jeopardize compliance. Research suggests that even temporary shortages of antihypertensive drugs might substantially reduce adherence in fragile healthcare systems (WHO, 2003) Consequently, policymakers must not disregard these underlying vulnerabilities.

Theoretical frameworks such as the Health Belief Model (Kam and Lee, 2024) offera conceptual framework: individuals assess perceived advantages against perceived obstacles, and younger persons may recognize fewer benefits from asymptomatic treatment.

These findings have substantial clinical, policy, and research implications. Healthcare practitioners ought to advocate for adherence screening among younger patients, particularly students. Tailored counselling, motivational interviewing, and technological interventions, like as SMS reminders or app-based trackers, may be more effective for this demographic.

From a policy perspective, Iraq’s healthcare system must prioritize the uninterrupted availability of medication, as it is crucial for sustaining elevated adherence rates. Universities and educational institutions ought to be incorporated as collaborators in fostering adherence by embedding hypertension education within student health services.

The research findings require the establishment of objective adherence criteria, such as pharmacy refill records or electronic monitoring, to validate self-reported adherence. Intervention studies should assess particular methodologies for young adults, including mobile health technologies and peer support frameworks. Qualitative study is essential to examine the psychological reasons that lead to insufficient adherence among students.

In conclusion, despite a high overall adherence rate to antihypertensive medicine in this Iraqi cohort, age emerged as the principal predictor, with younger patients—especially students—demonstrating markedly greater rates of non-adherence. These findings align with regional and global studies demonstrating that youth is consistently associated with poorer adherence to chronic disease treatments. Systemic support, including subsidised or complimentary access to medications, can greatly enhance overall adherence; however, behavioural factors exert a more substantial influence on vulnerable populations. Future methodologies must focus on youth-centered, behaviourally informed interventions to guarantee adherence over the lifetime.

## 5 Limitations

This research has some drawbacks. The cross-sectional method inhibits the determination of causation between predictors and adherence. Self-reported data may be susceptible to recall and social desirability biases, potentially leading to an underestimation of non-adherence. The study was conducted in a single province, perhaps limiting the generalizability of the findings to other regions of Iraq. Additionally, internal consistency was assessed using Cronbach’s alpha calculated for the overall instrument rather than for specific subdomains; therefore, the reported alpha value may not fully reflect internal consistency within individual constructs. A further limitation is that although the questionnaire was initially developed in English to align with international adherence frameworks, this approach may introduce subtle conceptual and cultural biases, despite the application of forward–backward translation and cultural adaptation procedures. Moreover, clinical indicators such as blood pressure regulation and comorbidities were not assessed, potentially providing further insights into adherence behavior. Notwithstanding these constraints, the study reveals essential sociodemographic and behavioral determinants of antihypertensive adherence.

## 6 Conclusion

This study provides context-specific evidence on factors associated with adherence to antihypertensive therapy in Iraq. Overall adherence was high; however, non-adherence was more frequently observed among younger patients, particularly students, and among individuals reporting limited engagement in self-care practices such as infrequent home blood pressure monitoring. Increasing age was associated with lower odds of non-adherence, while student status was associated with higher odds. Economic variables, medication cost, adverse effects, and provider communication were not significantly associated with adherence in this cohort, potentially reflecting the mitigating role of subsidized or low-cost medication access.

These findings identify population subgroups that may warrant focused attention in the design and evaluation of patient-centered support strategies. While causal relationships cannot be inferred from this cross-sectional analysis, the results may inform the development of targeted educational approaches, structured follow-up, and adherence-support initiatives. Future longitudinal and interventional studies are needed to confirm these associations and to assess the effectiveness of such strategies in improving medication adherence and blood pressure management in resource-constrained settings.

## Data Availability

The raw data supporting the conclusions of this article will be made available by the authors, without undue reservation.
