# Supplementary material for: Factors associated with non-adherence to antihypertensive therapy and blood pressure control in Iraq
Source: Front Pharmacol. 2026 May 21;17:1741906. doi: 10.3389/fphar.2026.1741906 (PMC13234455; doi:10.3389/fphar.2026.1741906)
Supplement: Supplementary file 1 [file Table1.docx]

**Supplementary Table S1. Content Validity Assessment of the Hypertension Adherence Questionnaire: Item-Level Expert Ratings and Content Validity Indices (I-CVI)**

| Domains | Item No. | Item Description | Expert 1 | Expert 2 | Expert 3 | I-CVI |
| --- | --- | --- | --- | --- | --- | --- |
| Domain 1: Demographic and Clinical Information | 1 | Age | 3 | 3 | 3 | 1.00 |
|  | 2 | Gender | 3 | 3 | 4 | 1.00 |
|  | 3 | Duration of hypertension | 4 | 3 | 3 | 1.00 |
|  | 4 | Presence of comorbidities | 3 | 3 | 3 | 1.00 |
|  | 5 | Current antihypertensive use | 3 | 4 | 4 | 1.00 |
|  | 6 | Type of medication | 4 | 4 | 4 | 1.00 |
|  | 7 | Place of residence | 4 | 4 | 4 | 1.00 |
|  | 8 | Employment status | 3 | 4 | 3 | 1.00 |
|  | 9 | Educational level | 3 | 3 | 4 | 1.00 |
| Domain 2: Medication Adherence | 10 | Frequency of medication intake | 4 | 4 | 4 | 1.00 |
|  | 11 | Missed medication last month | 3 | 4 | 3 | 1.00 |
|  | 12 | Number of missed doses | 3 | 4 | 3 | 1.00 |
|  | 13 | Reasons for missing medication | 4 | 3 | 4 | 1.00 |
|  | 14 | Altering dosage without consultation | 4 | 4 | 3 | 1.00 |
|  | 15 | Understanding provider instructions | 3 | 3 | 4 | 1.00 |
|  | 16 | Confidence managing medication | 4 | 3 | 4 | 1.00 |
| Domain 3: Knowledge and Beliefs | 17 | Belief medication controls BP | 3 | 3 | 4 | 1.00 |
|  | 18 | Concern about side effects | 3 | 4 | 3 | 1.00 |
|  | 19 | Received counseling | 3 | 3 | 4 | 1.00 |
|  | 20 | Importance of adherence | 3 | 4 | 4 | 1.00 |
|  | 21 | Belief lifestyle impact | 4 | 3 | 4 | 1.00 |
| Domain 4: Barriers and Challenges | 22 | Difficulty obtaining medication | 3 | 3 | 4 | 1.00 |
|  | 23 | Cost barrier | 3 | 3 | 4 | 1.00 |
|  | 24 | Side effects causing skipping | 3 | 3 | 3 | 1.00 |
|  | 25 | Family support | 3 | 3 | 3 | 1.00 |
|  | 26 | Provider communication | 3 | 3 | 4 | 1.00 |
|  | 27 | Use of alternative medicine | 3 | 3 | 3 | 1.00 |
| Domain 5: Self-Care and Follow-up | 28 | Frequency of BP monitoring | 4 | 4 | 4 | 1.00 |
|  | 29 | Attendance at appointments | 3 | 3 | 3 | 1.00 |
|  | 30 | Need for more counseling | 4 | 3 | 4 | 1.00 |

**S-CVI/Ave = 1.00‎**

**The Item-Level Content Validity Index (I-CVI) was calculated as the proportion of experts ‎assigning a rating of 3 or 4. The Scale-Level Content Validity Index (S-CVI/Ave) was ‎calculated as the average of all I-CVI values.‎**
